# Microbiota Perturbation or Elimination Can Inhibit Normal Development and Elicit a Starvation-Like Response in an Omnivorous Model Invertebrate

**DOI:** 10.1128/mSystems.00802-21

**Published:** 2021-08-24

**Authors:** Arturo Vera-Ponce de León, Benjamin C. Jahnes, Alejandro Otero-Bravo, Zakee L. Sabree

**Affiliations:** a Department of Evolution, Ecology and Organismal Biology, The Ohio State University, Columbus, Ohio, USA; University of Connecticut

**Keywords:** *Periplaneta americana*, germfree, gnotobiotic, microbiome

## Abstract

Omnivorous animals, including humans, harbor diverse, species-rich gut communities that impact their growth, development, and homeostasis. Model invertebrates are broadly accessible experimental platforms that enable linking specific species or species groups to host phenotypes, yet often their specialized diets and distinct gut microbiota make them less comparable to human and other mammalian and gut communities. The omnivorous cockroach Periplaneta americana harbors ∼4 × 10^2^ bacterial genera within its digestive tract and is enriched with taxa commonly found in omnivorous mammals (i.e., *Proteobacteria, Bacteroidetes*, and *Firmicutes*). These features make *P. americana* a valuable platform for identifying microbe-mediated host phenotypes with potential translations to mammals. Rearing *P. americana* insects under germfree conditions resulted in prolonging development time by ∼30% and an up to ∼8% reduction in body size along three dimensions. Germfree rearing resulted in downregulation of gene networks involved in growth, energy homeostasis, and nutrient availability. Reintroduction of a defined microbiota comprised of a subset of *P. americana* commensals to germfree insects did not recover normal growth and developmental phenotypes or transcriptional profiles observed in conventionally reared insects. These results are in contrast with specialist-feeding model insects (e.g., *Drosophila*), where introduction of a single endemic bacterial species to germfree condition-reared specimens recovered normal host phenotypes. These data suggest that understanding microbe-mediated host outcomes in animals with species-rich communities should include models that typically maintain similarly diverse microbiomes. The dramatic transcriptional, developmental, and morphological phenotypes linked to gut microbiome status in this study illustrates how microbes are key players in animal growth and evolution.

**IMPORTANCE** Broadly accessible model organisms are essential for illustrating how microbes are engaged in the growth, development, and evolution of animals. We report that germfree rearing of omnivorous *Periplaneta americana* cockroaches resulted in growth defects and severely disrupted gene networks that regulate development, which highlights the importance of gut microbiota in these host processes. Absence of gut microbiota elicited a starvation-like transcriptional response in which growth and development were inhibited while nutrient scavenging was enhanced. Additionally, reintroduction of a subset of cockroach gut bacterial commensals did not broadly recover normal expression patterns, illustrating that a particular microbiome composition may be necessary for normal host development. Invertebrate microbiota model systems that enable disentangling complex, species-rich communities are essential for linking microbial taxa to specific host phenotypes.

## INTRODUCTION

Animal digestive tracts are inhabited by microbial communities that are comprised of a small to a large number of species that can contribute to dietary processing and nutrient provisioning, pathogen protection, and detoxification of harsh and xenobiotic compounds in the diet (1). Additionally, gut microbiota can modulate expression of genes involved in growth, development, and homeostasis in the gut of the animal host (2–5). Animal model systems, especially those that can survive without their gut bacteria, enable linking microbiota to host outcomes (6, 7). Axenic or “germfree” animals reared and maintained under sterile conditions have provided a powerful platform for assessing the impact of bacteria on gut physiology and metabolism (4, 5, 8–11). Germfree invertebrates (e.g., flies, honeybees, and mosquitoes) (4, 10, 12) have many of the advantages of similarly husbanded mammals (e.g., mice and pigs) and are often cheaper to maintain and have more rapid generation times. The specialized diets and relatively reduced bacterial communities of some model invertebrates (Drosophila melanogaster, 1 to 30 bacterial species; Apis mellifera, 8 to 10 bacterial species [13, 14]) limit their applicability to animals with complex microbial communities like humans and other omnivorous mammals. In contrast, cockroaches, such as the peridomestic American cockroach, Periplaneta americana (Blattodea: Blattidae), are also opportunistic omnivores and possess a diverse gut bacterial population (∼4 × 10^2^ genera) (15, 16). The bacterial community in *P. americana* is predominated by members of the *Bacteroidetes*, *Firmicutes*, and *Proteobacteria* (15, 17, 18), which are also commensals in mammals. Germfree *P. americana* cockroaches reared under sterile conditions exhibited prolonged development, smaller body size, and digestive dysmorphias that included flaccidity, lack of pigmentation, and reduced lateral infoldings at the luminal surface (19), suggesting that its gut commensals contribute to normal development.

To identify host genes and gene networks whose expression was impacted by the elimination of the gut bacterial community in *P. americana*, we quantified gene expression in the midgut and hindgut in the presence (conventionally reared [Conv]) and absence (germfree [GF]) of commensal bacteria. Additionally, we introduced a polyspecific assemblage of cultivable *P. americana* commensals comprised of a subset of species representative of taxa typically dominant in the gut (i.e., *Bacteroidetes* and *Firmicutes*) to GF *P. americana*, resulting in gnotobiotic (GN) insects. Comparative developmental, morphological, and transcriptional analyses of these treatments further illustrates how the presence and composition of commensal microbial communities impact host growth and development.

## RESULTS

### Germfree and gnotobiotic insects exhibited significant developmental delays and growth deficiencies.

**FIG 1:**
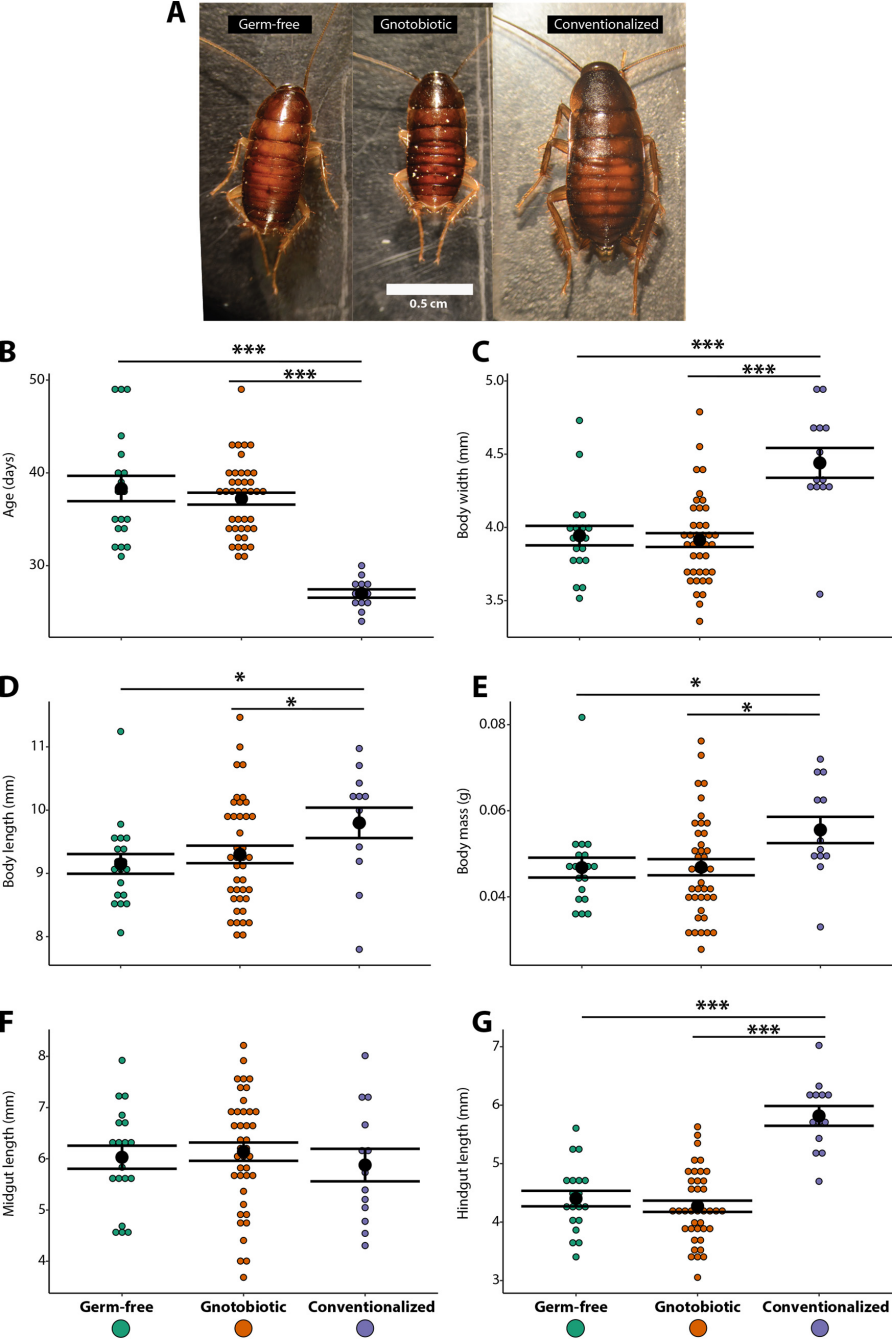
Phenotypic and morphological alterations by bacterial dysbiosis in *P. americana* insects. (A) Fifth-instar *P. americana* insects reared with no bacteria (germfree [GF]), reared with a subset of the bacterial community (gnotobiotic [GN]), and conventionalized (Conv). (B) Duration (stadium) of individuals to complete the fifth-instar molting. (C to E) *P. americana* body width (C), length (D), and mass (E) measurements. (F and G) *P. americana* midgut (F) and hindgut (G) lengths. Black dots and bars represent the mean ± standard error of the mean (SEM). Asterisks indicate significant differences between treatments (*, *P < *0.05; ***, *P* <0.001).

Molting rate and growth (i.e., body width, length, and mass and midgut and hindgut lengths from fifth-instar insects), as measures of development, were substantially impacted by the presence and composition of the gut microbiome in *P. americana* (Fig. 1A). Developmental time was prolonged by up to 10 days (∼30% increase) in GF and GN insects in comparison to that of Conv insects (Kruskal-Wallis *P* = 0.0001) (Fig. 1B and Table 1), but no differences were appreciated between GF and GN insects (Fig. 1B and Table 1). GF and GN insects had decreased body width (Kruskal-Wallis *P* = 0.0002), length (Kruskal-Wallis *P* = 0.049), and mass (Kruskal-Wallis *P* = 0.033) (Fig. 1C to E and Table 1), but no GF versus GN differences were observed (Kruskal-Wallis *P* > 0.05). While midgut length was not significantly different between treatments (Kruskal-Wallis *P* = 0.66), GF and GN hindgut lengths were significantly smaller (Kruskal-Wallis *P* = 1.3 × 10^−6^) (Fig. 1F and G and Table 1).

**TABLE 1:** Microbial status impacts several measures of insect growth and development^*a*^

Trait	Treatment	Mean	SE
Ootheca to fifth instar (days)	GF	38.32	1.37
	GN	37.23	0.64
	Conv	27	0.45
Body width (mm)	GF	3.94	0.07
	GN	3.91	0.05
	Conv	4.44	0.10
Body length (mm)	GF	9.15	0.16
	GN	9.30	0.14
	Conv	9.80	0.24
Body mass (g)	GF	0.05	0.002
	GN	0.05	0.002
	Conv	0.06	0.003
Midgut length (mm)	GF	6.03	0.22
	GN	6.14	0.18
	Conv	5.88	0.32
Hindgut length (mm)	GF	4.40	0.13
	GN	4.27	0.10
	Conv	5.82	0.17

a Mean and standard error (SE) values are reported for germfree (GF; *n* = 19), gnotobiotic (GN; *n* = 40), and conventionalized (Conv; *n* = 13) insects.

### *De novo* cockroach gut transcriptome assembly and annotation.

High-resolution transcriptome profiling of GF, GN, and Conv *P. americana* midgut and hindguts (see Data Set S1 in the supplemental material for main statistics) was performed to reveal gene-level changes linked to these treatments. As no gene models were associated with the *P. americana* genome when these analyses were conducted (Autumn 2019), a *de novo* assembly of all sequence reads was performed. Transcriptome assembly yielded 369,082 gene models and 554,155 isoforms (Table S1), and 12% of these transcripts matched an annotated product in at least one of the reference databases used (UniProt, Pfam, KEGG). BUSCO analysis estimated a 93.6% transcriptome completeness of the nonredundant contigs containing all the *P. americana* gut transcripts. Additionally, 1,008 putative bacterial genes were annotated in the *P. americana* genome and ultimately excluded from these analyses. After bacterial decontamination, 553,147 assembled isoforms (Table S1) were used for transcript quantification and differential expression analysis. Only 8,352 of these transcripts were differentially expressed (fold change ≥ 1.5, false discovery rate [FDR] ≤ 0.05) in the GF, GN, and Conv insects of both midgut and hindgut and showed a homologous sequence in at least one of the public databases used for annotation (Table 2).

**TABLE 2:** Microbial status impacts differential gene expression in *P. americana* hindgut and midgut tissues^*a*^

Tissue	Treatment comparison	No. of differentially expressed transcripts (FC ≥ 1.5, FDR ≤ 0.05)^*b*^
Midgut	GF vs GN	827
	GF vs Conv	1,349
	GN vs Conv	3,027
Hindgut	GF vs GN	1,360
	GF vs Conv	268
	GN vs Conv	1,521

a All transcripts have homologous annotations with coding elements present in at least one of the reference databases. GF, germfree; GN, gnotobiotic; Conv, conventionally reared.

b Fold change (FC) and false discovery rate (FDR) values were obtained by DESeq2 analysis.

10.1128/mSystems.00802-21.1 TABLE S1 General features of *P. americana* genome and gut transcriptome. Download Table S1, DOCX file, 0.01 MB. Copyright © 2021 Vera-Ponce de León et al. 2021 Vera-Ponce de León et al. https://creativecommons.org/licenses/by/4.0/ This content is distributed under the terms of the Creative Commons Attribution 4.0 International license.

10.1128/mSystems.00802-21.5 DATA SET S1 Main statistics of RNA-Seq from *P. americana* gut. Download Data Set S1, XLSX file, 0.01 MB. Copyright © 2021 Vera-Ponce de León et al. 2021 Vera-Ponce de León et al. https://creativecommons.org/licenses/by/4.0/ This content is distributed under the terms of the Creative Commons Attribution 4.0 International license.

### Gut region and microbiome status defined *P. americana* expression profiles.

**FIG 2:**
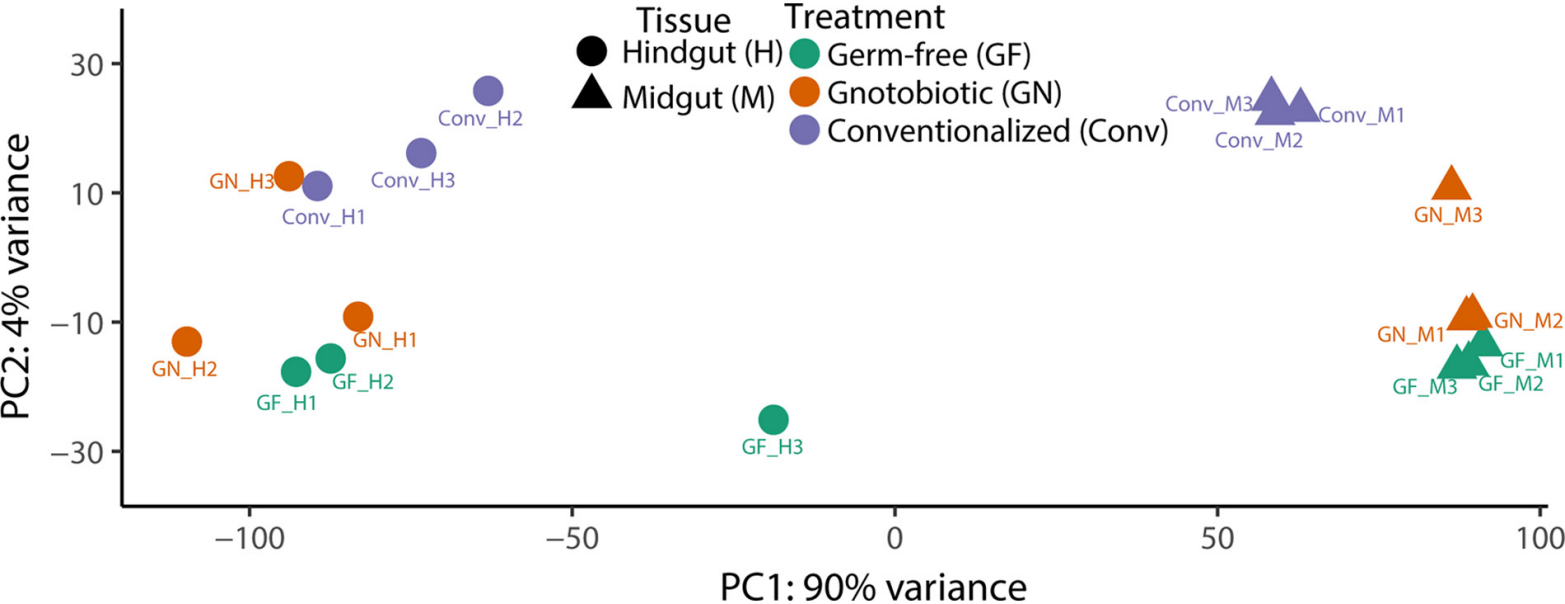
Transcriptomic analysis revealed alterations in the presence and absence of bacteria in *P. americana* midgut and hindgut. Principal-component analysis (PCA) of RNA-Seq transcripts after DESeq2 normalization. Replicate (*n* = 3) transcriptome libraries from hindgut (circles) and midgut (triangles) tissues from conventionalized (red), gnotobiotic (green), and germfree (blue) insects are depicted.

In general, the transcriptomes differed sharply along gut region (midgut and hindgut) and treatment (GF, GN, and Conv) axes as principal-component analysis (PCA) analyses revealed that gut region (i.e., midgut and hindgut; *x* axis, 90% variance) and microbiome status (i.e., GF, GN, and Conv; *y* axis, 4% variance) defined transcriptome placement within ordination space (Fig. 2). A linear model using all expressed isoforms showed significant differences in the expression of the transcripts among microbiome status (analysis of variance [ANOVA] *P = *9.11e−11) (Table S2). This confirms that changes in microbiome status, by either elimination (GF) or gnotobiosis (GN), altered host gut transcriptional expression profiles.

10.1128/mSystems.00802-21.2 TABLE S2 Summary of the analysis of variance (ANOVA) on the expression values of differentially expressed transcripts in the *P. americana* midgut and hindgut (gut region) under three different microbiome status conditions (i.e., germfree, gnotobiotic, and conventionalized). Download Table S2, DOCX file, 0.01 MB. Copyright © 2021 Vera-Ponce de León et al. 2021 Vera-Ponce de León et al. https://creativecommons.org/licenses/by/4.0/ This content is distributed under the terms of the Creative Commons Attribution 4.0 International license.

**FIG 3:**
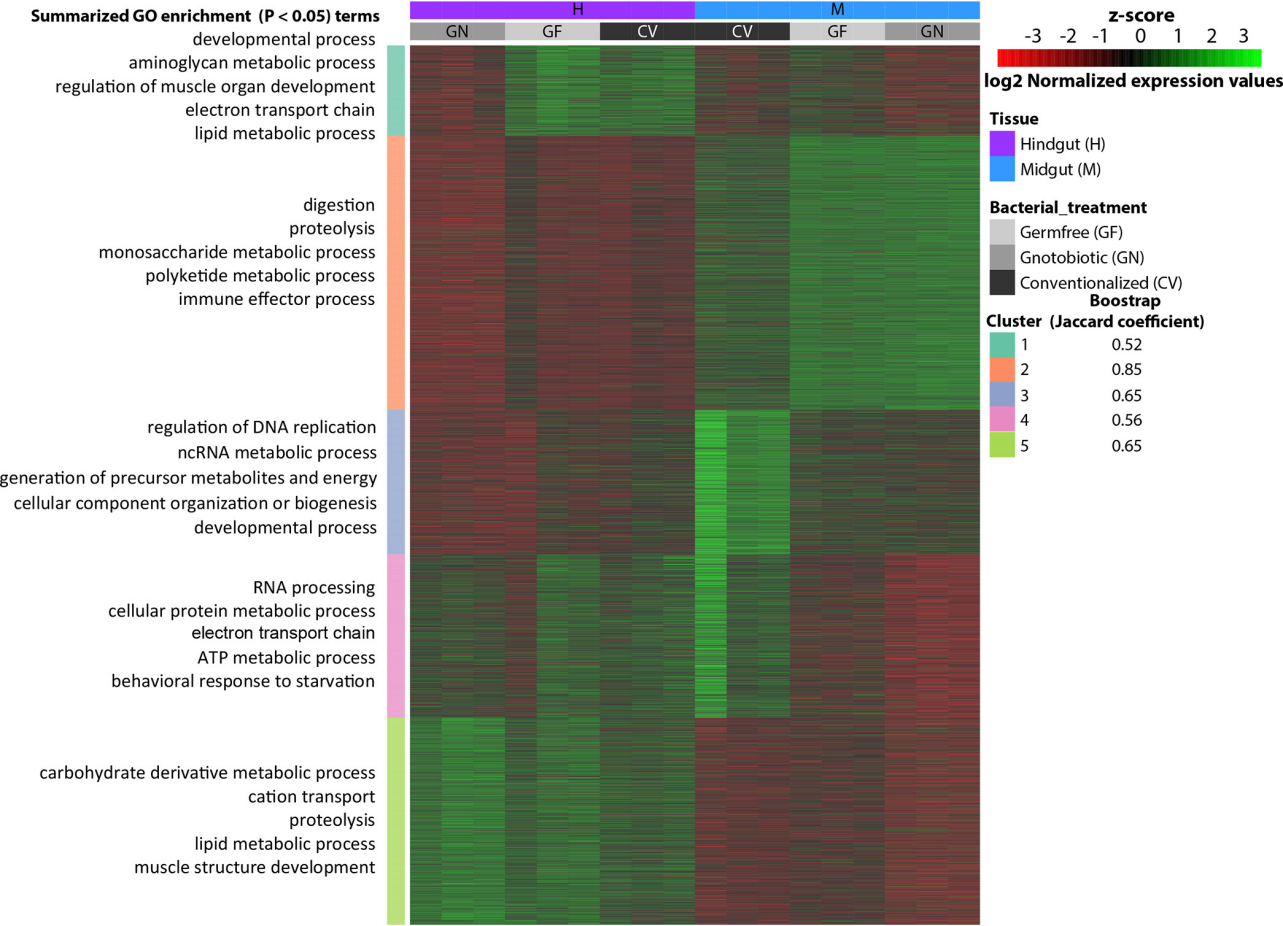
Hierarchical clustering of differentially expressed genes from *P. americana* gut. The heat map shows the Z-score of the RNA-Seq counts after DEseq2 normalization of all treatments (GF, GN, and Conv) in both midgut and hindgut. Gene ontologies of the five resulting clusters were analyzed by GOseq and summarized using REVIGO (see Materials and Methods). REVIGO annotation of the main GO biological process categories in each cluster are shown. See Data Set S2 in the supplemental material for the complete list of GO terms in each cluster.

To identify transcripts whose expression was specifically altered by microbiome status, a hierarchical clustering (HC) of the 5,258 unique, annotated, and differentially expressed transcripts among all treatments was performed. HC resulted in 54% of the transcripts forming two clusters defined by the midgut and hindgut regions (clusters 2 and 5) (Fig. 3; Data Set S2) and the remaining transcripts forming three clusters defined by both gut region and microbiome status (clusters 1, 3, and 4) (Fig. 3; Data Set S2). Bootstrap analysis of HC showed Jaccard similarity coefficient values higher than 0.5 in all clusters (Fig. 3), suggesting that the five clusters reflect stable patterns in the data (20). Gene ontology (GO) term enrichment analysis of transcripts in clusters 1, 3, and 4 revealed that changes in the microbiome status (i.e., GF and GN) reduced the expression and dramatically affected host processes that spanned gene families involved in cellular and organ growth, tissue development and homeostasis, lipid transport, electron transport chain, regulation of DNA replication, RNA metabolism, protein metabolic process, generation of precursor metabolites and energy ATP metabolic process, and behavioral response to starvation (Fig. 3; Data Set S2). This indicates that both microbiome elimination (GF) and gnotobiosis (GN) alter genes directly involved in the correct development and energetic metabolism in the *P. americana* gut.

10.1128/mSystems.00802-21.6 DATA SET S2 Differentially expressed genes of *P. americana* gut under different bacterial treatments after hierarchical clustering, GO enrichment, and REVIGO analyses. Sheets labeled as No.Cluster show all transcripts and annotations from each cluster. Sheets labeled as No.Cluster.Revigo show all transcripts after GO enrichment and REVIGO summarizing (see Materials and Methods). The log2 fold change and FDR (adjusted *P* value) of all differentially expressed transcripts (log2 fold change ≥ 0.5 and FDR ≤ 0.05) after DESeq2 analysis in all transcriptome comparisons (i.e., midgut/hindgut and GF, GN, and Conv treatments) are shown. Download Data Set S2, XLSX file, 4.7 MB. Copyright © 2021 Vera-Ponce de León et al. 2021 Vera-Ponce de León et al. https://creativecommons.org/licenses/by/4.0/ This content is distributed under the terms of the Creative Commons Attribution 4.0 International license.

### IIS and TOR signaling cascades were negatively affected by bacterial elimination and gnotobiosis.

**FIG 4:**
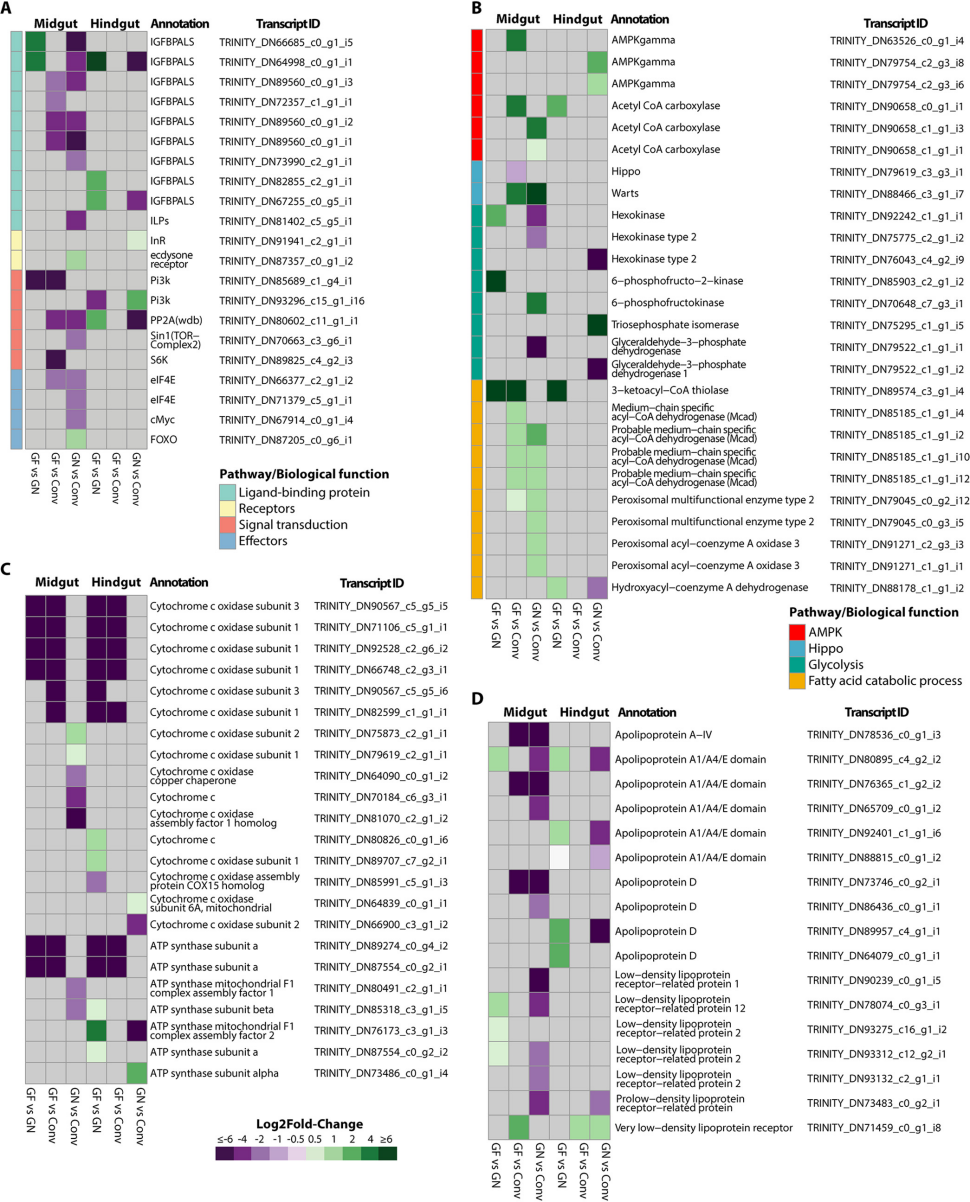
Germfree and gnotobiotic treatments produce changes in the expression of genes involved in development and energy homeostasis of *P. americana* midgut and hindgut. Heat maps showing differentially expressed values from genes involved in the IIS-TOR pathways (A) and AMPK, Hippo, glycolysis, and fatty acid catabolism metabolic pathways (B) and encoding cytochrome oxidase and ATP synthase (C) and lipoproteins (D) in *P. americana* midgut and hindgut under the three different treatment comparisons, conventionalized versus gnotobiotic (Conv vs GN), germfree versus conventionalized (GF vs Conv), and gnotobiotic versus conventionalized (GN vs Conv). All isoform identifiers and annotation and expression values associated with these pathways are described in Data Set S3 in the supplemental material. The color range in heat maps indicates the log_2_ fold change. Gray boxes indicate no differential expression observed after DESeq2 analysis (FDR > 0.05).

Alterations in the insulin/insulin-like growth factor signaling (IIS) and target of rapamycin (TOR) pathway genes usually affect normal cellular growth, development, body size, and energy and nutrient availability (2, 4, 21). Gene families and GO terms associated with these pathways were detected as enriched (GO term *P* value < 0.05) in clusters (i.e., 1, 3, and 4) where the expression values were altered by changes in the microbiome status (Fig. 3; Data Set S2). Insulin growth factor binding protein acid-labile subunits (IGFBP-ALS) promote the stability of insulin growth factors that promote cellular growth and tissue development in metazoans (22, 23), and several IGFBP-ALS isoforms were downregulated in midgut GF and GN treatments (Fig. 4A; Data Set S3). While no treatment-specific expression patterns were observed in the insulin-like receptor (InR) (Data Set S3), genes coding for various parts of the cognate signal transduction phosphorylation cascade of the IIS pathway (i.e., Pi3K and PP2A [Fig. 4A] and RAS [Data Set S3]) were also downregulated in GF midguts. TOR pathway effectors, eukaryotic translation initiation factor 4E (eIF4E), and the ribosomal protein kinase S6K (Fig. 4A; Data Set S3) also exhibited reduced expression in GF insects, which could negatively impact TOR pathway-regulated ribosome biogenesis, protein biosynthesis, and tissue growth functions.

10.1128/mSystems.00802-21.7 DATA SET S3 Expressed transcripts of different metabolic pathways in *P. americana* midgut and hindgut under different bacterial treatments: germfree, gnotobiotic, and conventionalized. “Total” suffixed sheets show the total expression values of each transcript isoform after DEseq2 analysis. “DEG” suffixed sheets show the differentially expressed transcript isoforms (log2 fold change ≥ 0.5 and FDR ≤ 0.05) after DESeq2 analysis. Columns show Trinity transcript ID, annotation, log2 fold change, *P* values, and FDR (P-adjust value) after Benjamini-Hochberg correction. GF vs GN, germfree versus gnotobiotic; GF vs Conv, germfree versus conventionalized; GN vs Conv, gnotobiotic versus conventionalized. The last letter indicates the tissue midgut (M) or hindgut (H). Example: log2foldchange.GFvsGNM = log2 fold change of germfree versus gnotobiotic comparison in the midgut. Download Data Set S3, XLSX file, 0.8 MB. Copyright © 2021 Vera-Ponce de León et al. 2021 Vera-Ponce de León et al. https://creativecommons.org/licenses/by/4.0/ This content is distributed under the terms of the Creative Commons Attribution 4.0 International license.

Gnotobiotic (GN) *P. americana* insects exhibited IIS-TOR pathway expression patterns distinct from those of Conv insects, suggesting that the commensal assemblage was incapable of recovering Conv-level IIS-TOR pathway expression (Fig. 4A). In addition to eIF4, the Myc TOR effector and its activator Akt1 were downregulated in GN midgut tissues (Fig. 4A; Data Set S3), which can contribute to prolonged growth (Fig. 1A to D) due to reduced ribosome biogenesis and protein translation (24). Additionally, upregulation of the TOR-induced negative regulator FOXO in GN midgut tissues (Fig. 4A), which is involved in protein synthesis inhibition and organ size regulation (21, 25), may also explain the observed phenotypes in GN insects (Fig. 1A to D).

### Microbiome removal and gnotobiosis elicit energy stress in *P. americana* gut tissues.

Genes involved in responding to metabolic stress resulting from energy deprivation also exhibited altered expression patterns (Fig. 3 and Fig. 4B). The intracellular AMP sensing protein, 5′-AMP-activated protein kinase (AMPK) (26–28), and the Hippo pathway negative regulator (26, 27) Warts kinase were among metabolic stress-relevant genes that were upregulated in GF midgut tissues (Fig. 4B; Data Set S3). Downregulation of mitochondrial respiration and energy production enzymes, ATP synthase, and cytochrome *c* oxidase (29) in GF midgut and hindgut tissues further linked bacterial absence to a possible altered energy homeostatic condition (Fig. 3 and 4C; Data Set S3). Likewise, under energy stress, AMPK activity stimulates fatty acid oxidation that can liberate precursors for energy production (30). Several catabolic fatty acid oxidation genes and their isoforms (medium-chain-specific acyl coenzyme A [acyl-CoA] dehydrogenase [MCAD], 3-ketoacyl-CoA thiolase [ACAA2], and peroxisomal multifunctional enzyme type 2 short-chain dehydrogenase) (31, 32) were upregulated in the GF midgut tissues (Fig. 4B; Data Set S3). Additionally, multiple lipoprotein-encoding isoforms were downregulated in GF midgut tissues (Fig. 4D; Data Set S3), and this phenotype has been linked to energy stress (30). Contrary to expectations, GN insects appeared to be under greater energy stress than GF insects, given the enhanced upregulation of Warts kinase and catabolic fatty acid genes (e.g., MCAD and peroxisomal acyl-coenzyme A oxidase 3) (Fig. 4B) and downregulation of lipoprotein isoforms (Fig. 4D). Moreover, dysregulation of 6-phosphofructokinase, hexokinase, and glyceraldehyde-3-phosphate dehydrogenase and ATP synthase genes, commonly associated with energy acquisition by glycolysis (29), further describes a state of energy stress in GN insects (Fig. 4B).

### Germfree and gnotobiotic *P. americana* insects displayed altered expression of genes involved in carbohydrate catabolism in the gut.

**FIG 5:**
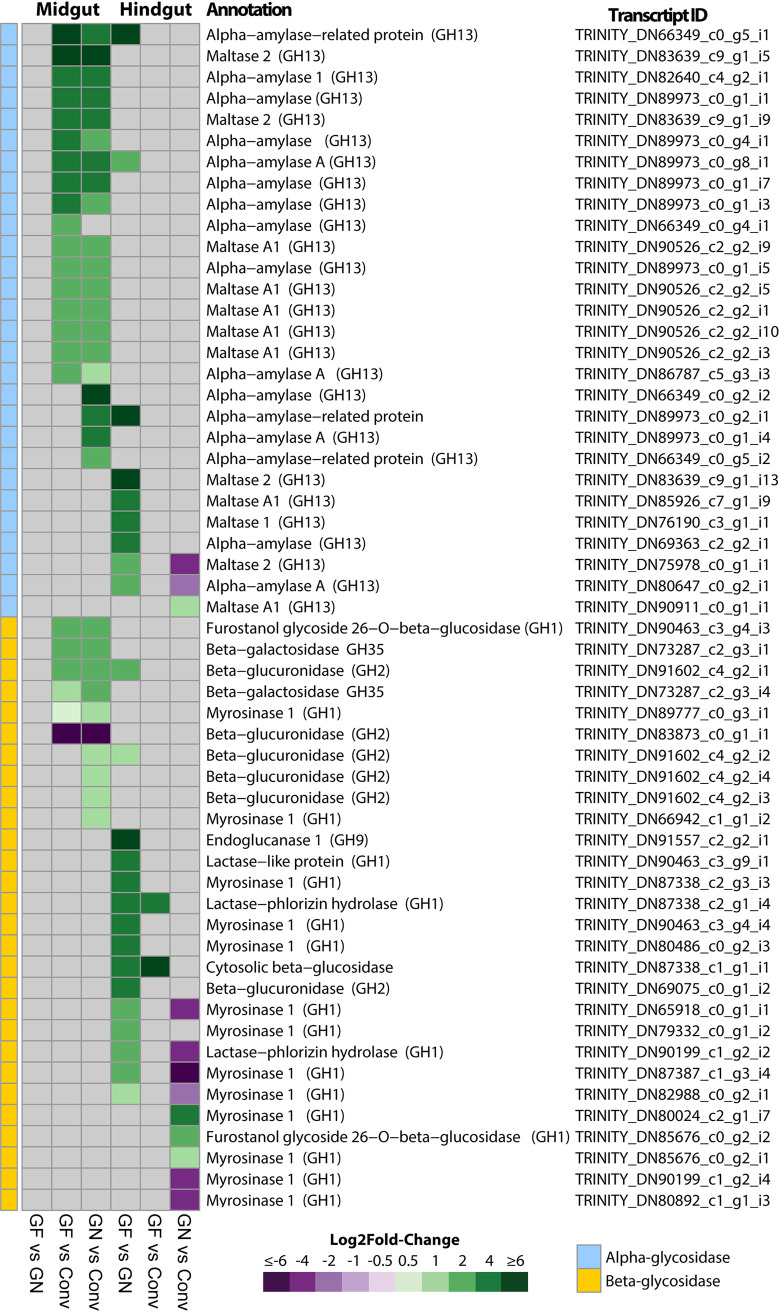
Changes in the expression of genes involved in carbohydrate catabolism in *P. americana* midgut and hindgut tissues. A heat map shows differentially expressed values of genes encoding glycoside hydrolases (GH). The color range in heat maps indicates the log_2_ fold change in *P. americana* midgut and hindgut under the three different treatments comparison, conventionalized vs gnotobiotic (Conv vs GN), germfree vs conventionalized (GF vs Conv) and gnotobiotic vs conventionalized (GN vs Conv). All isoform identifiers, annotation, and expression values associated with this pathway are described in Data Set S3 in the supplemental material. The color range in heat maps indicates the log_2_ fold change. Gray boxes indicate no differential expression observed after DESeq2 analysis (FDR > 0.05).

Differential expression of host glycoside hydrolase (GH) genes involved in carbohydrate catabolism in digestive tissues was examined. Upregulation of GH genes in the gut could reflect host attempts to use endogenous enzymes to liberate assimilable carbohydrates in response to energy stress. Several α-amylase, maltase (GH13), and β-glycosidase (GH1, GH2, and GH35)-encoding isoforms were upregulated in the GF midgut tissues (Fig. 5; Data Set S3). Additionally, two β-glycosidases were also upregulated in GF hindgut tissues (Fig. 5; Data Set S3). Although bacteria comprising the inoculum used to generate GN insects encoded polysaccharolytic enzymes (33), many of the GHs that were upregulated in the GF midgut tissues were similarly upregulated in the GN midgut tissues, with a few additional alpha-amylase and beta-glucoronidase isoforms as well (Fig. 5; Data Set S3). In the hindgut tissues, a suite of alpha- and beta-glycosidase isoforms that were largely distinct from those expressed in the midgut tissues exhibited decreased expression (Fig. 5; Data Set S3).

### Polysaccharolytic isolates are at high abundance in the gnotobiotic insects.

**FIG 6:**
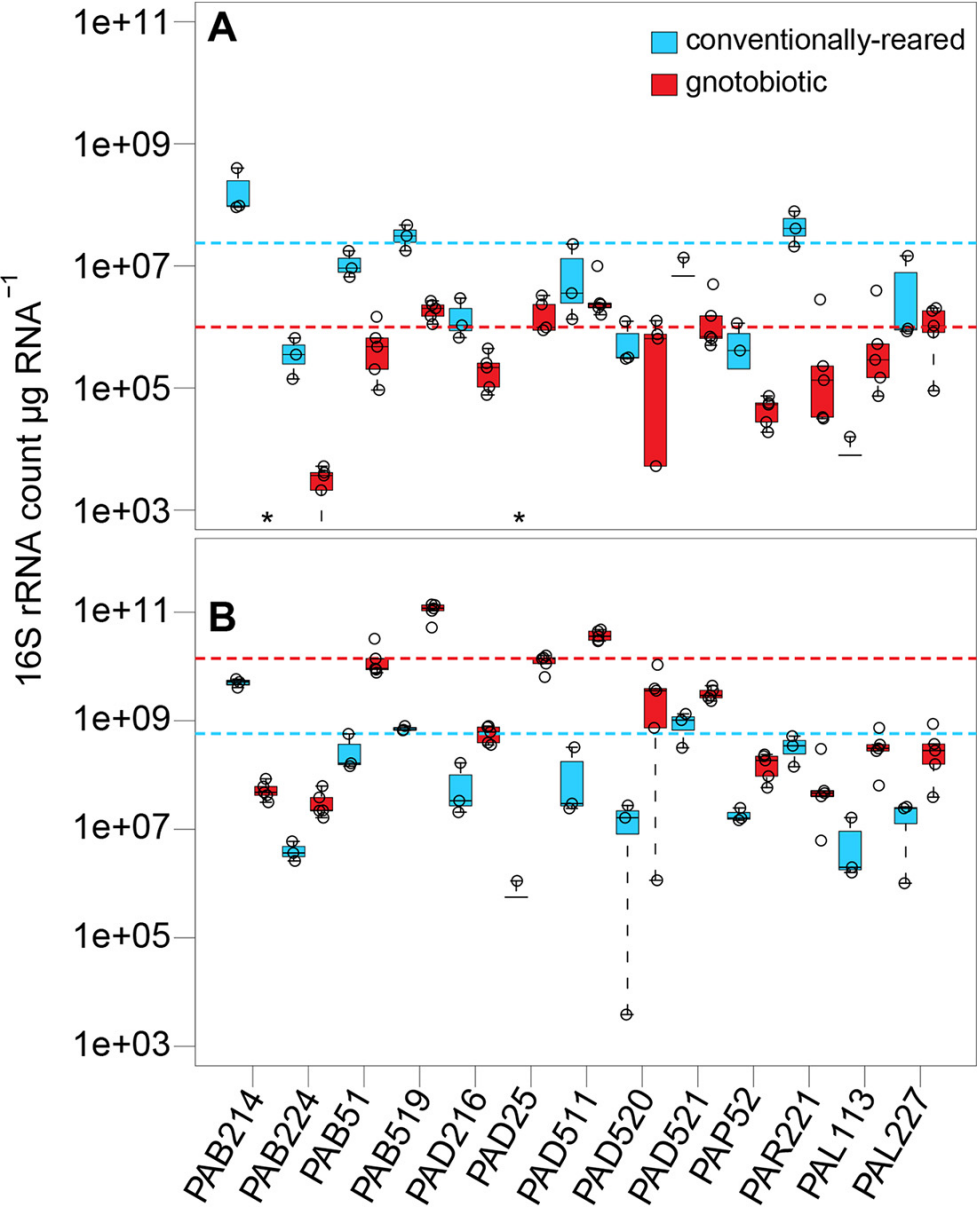
Gut commensal prevalence of typical gut microbiota in *P. americana* midgut and hindgut tissues. Isolate-specific primers were used to detect the abundance of *P. americana* gut commensals in the midgut (A) and hindgut (B) tissues of gnotobiotic (red) and conventionally reared (blue) insects. Dashed lines represent the mean abundances for all isolates in gnotobiotic (red) and conventionally reared (blue) insects.

Quantitative reverse transcription PCR (RT-qPCR) targeting isolate-specific 16S rRNA detected the presence of all bacteria in the hindgut of both Conv and GN insects (Fig. 6B). In contrast, in the GN midgut, the isolate *Bacteroides* sp. strain PAB214 was not detected (Fig. 6A). Likewise, the isolate *Dysgonomonas* sp. strain PAD25 was not found in the Conv midgut and was present only in a single pool in the Conv hindgut (Fig. 6A and B). Nonetheless, this evidence suggests that most of the isolates used for gnotobiosis can successfully colonize both midgut and hindgut.

The four most abundant (78% of the total isolate bacterial population) isolates that colonized the GN midgut showed amylase activity *in vitro*, with zones of clearing formed in a starch degradation assay on solid agar medium (33). Likewise, higher proliferation of the cellulolytic *Bacteroidetes* strains PAB51, PAB519, and PAD25 (33) was observed in the GN hindgut than in Conv insects (Fig. 6B). This suggests that a large proportion of the bacterial community used in the GN treatment have the potential to compete with the host for dietary energy sources (e.g., simple sugars, starch, and cellulose).

## DISCUSSION

### Eliminating gut microbiota in *P. americana* insects recapitulates energy and nutrient starvation responses observed in other insects and mice. (i) IIS and TOR pathways.

Detailed expression profiling of *P. americana* mid- and hindgut tissues supported treatment-defined physiological observations where elimination and/or alteration of the bacterial community resulted in treatment-specific expression of gene networks involved in insulin-insulin signal (IIS), target of rapamycin (TOR), and other nutrient sensing and growth and development pathways. Similarly, elimination of bacteria in *Drosophila* and Apis mellifera also reduced the expression of genes involved in these pathways, which was correlated with prolonged growth periods, smaller body sizes, and alterations in energy homeostasis (2, 4, 34). *Drosophila* flies experiencing nutrient and energy stress exhibited similar expression profiles, including decreased Pi3K and RAS signaling in various peripheral tissues (e.g., the prothoracic gland and fat bodies) (24, 28), reduced IGFBP-ALS expression (22, 28), and increased AMPK and Warts kinase expression (26, 30). Likewise, IGFBP-ALS and S6K deletions in mice resulted in delayed development in postnatal mice (23) and small-body phenotypes (35), respectively.

### (ii) AMPK.

AMPK upregulation is notable as it correlates with the absence of bacterium-derived short-chain fatty acids (e.g., acetate, propionate, and butyrate) in GF mice (36), which serve as a major energy source for intestinal epithelial cells. In a related study, cell cultures deprived of glucose exhibited elevated AMPK and Warts kinase expression (26, 27). Given that AMPK can activate Warts, which can subsequently inhibit the Hippo effectors Yorki and Mads, resulting in reduced cell proliferation (26, 27), energy-expensive activities appear limited in GF gut tissues. Additionally, upregulation of AMPK by Warts often correlates with reduced ATP synthesis and decreasing carbohydrate availability (27, 37). This result agrees with the severe downregulation in respiration and energy production genes, ATP synthase, and cytochrome *c* oxidase (29), observed in mid- and hindgut GF tissues. Similarly, reduced mitochondrial respiration and ATP synthase activity were observed in colonocytes of GF mice, which was linked to energy stress (36).

### (iii) Fatty acid and carbohydrate catabolism.

Increased AMPK expression, which was observed in germfree insects, has been linked to mobilization of stored fatty acids. Gut microbiota are primary producers of short-chain fatty acids, and mobilization of stored fatty acids may reflect a host response to their absence in germfree insects. Several genes involved in fatty acid oxidation were upregulated in GF *P. americana* tissues, and upregulation of fatty acid oxidative genes has been reported in the midgut of germfree larvae of the mosquito Aedes aegypti (10). Upregulation of AMPK and fatty acid catabolism in germfree mice were correlated with leaner-bodied individuals with lower body masses, even when feeding on a high-calorie-content diet, relative to conventionalized mice (30). This agrees with our results where GF *P. americana* insects were reduced in size and body mass even when fed a high-nutrient diet (Fig. 1A to E). Additionally, reduced lipoprotein lipase (LPL) in the intestinal epithelium was correlated with leaner phenotypes in mice (30), and LPL orthologues in GF *P. americana* insects were similarly downregulated (see Data Set S3 in the supplemental material). We also expected that energy stress would elicit upregulation of endogenous carbohydrate degradation enzymes to increase host access to dietary nutrients, and several amylase, myrosinase, beta-galactosidase, and beta-glucuronidase genes were upregulated in GF *P. americana* mid- and hindguts (Fig. 5; Data Set S3). Upregulation of β-glycosidases in GF *P. americana* hindguts was notable, as glycosidase expression in these tissues in insects is not well documented (38). Similarly, nutrient-deprived *Drosophila* flies exhibited upregulation of starch-degrading (e.g., amylase GH13) genes as well (39, 40).

### *P. americana* relies upon a specific microbial assemblage for successful development.

Elimination of gut bacteria from *P. americana* via germfree rearing resulted in prolonged development and reduced physiological growth along several metrics, yet oral reintroduction of a select group of *P. americana* gut commensals did not recover normal phenotypes (Fig. 1A to G; Table 1). These results were unexpected, as mono- and polycultural inoculation with typical or atypical gut bacteria could recover normal phenotypes in fruit flies and honeybees (2–4, 41, 42). Transcriptional profiling was used to illustrate how alterations of the gut microbial community affected major host gene networks involved in growth and development. Broadly, the transcriptional profiles of gnotobiotic (GN) *P. americana* were, especially in the case of the midgut samples, more similar to the GF treatments (Fig. 3). Additionally, genes involved in or coding for IIS-TOR, AMPK, glycolysis, fatty acid catabolic processes, cytochrome oxidases and ATP synthases, lipoproteins, and glycosyl hydrolases in GN midgut tissues exhibited differential expression patterns like those observed in GF tissues (Fig. 4A to D and 5). GN hindgut tissues exhibited expression patterns that were similar to those of GF hindgut tissues (Fig. 3). Transcriptional profiles from GN tissues exhibited signatures of energy and nutrient stress like those observed in GF tissues. This suggests that the presence of the select bacterial commensals in *P. americana* gut magnifies expression of many of the same genes and/or isoforms as GF treatments and alters the expression of additional loci (Fig. 3). This differs from what was previously observed in other gnotobiotic invertebrate models (2–4, 10). For example, inoculation of gnotobiotic *Drosophila* flies with the commensal Acetobacter pomorum or Lactobacillus plantarum resulted in wild-type level IIS-TOR pathway activation (2, 3), indicating that these isolates alone could promote larval development in *Drosophila.* A. pomorum also downregulated expression of the organ growth negative regulator FOXO, which was correlated with restoring the normal developmental phenotype in D. melanogaster larvae (2), yet FOXO was upregulated in GN *P. americana* midgut tissues, indicating that these commensals could not alleviate TOR inhibition (Fig. 4A). As *Drosophila* and *P. americana* insects had analogous IIS-TOR pathway responses following germfree rearing (2, 3), dysregulation of the IIS-TOR pathway in the latter by typical *P. americana* gut microbiome members was unexpected, given the restoration of wild-type expression in similar gnotobiosis experiments in *Drosophila.*

AMPK, Hippo, glycolysis, and fatty acid catabolism gene networks responded similarly where introduction of the commensal bacterial community in *P. americana* magnified expression patterns observed in GF insects (i.e., two acetyl-CoA carboxylase isoforms and Warts kinase), and the altered expression of additional genes, including those involved in glycolysis (Fig. 4B) and lipoprotein production (Fig. 4D), further support an energy/nutrient-stressed condition. As in GF insects, GN insects exhibited elevated fatty acid catabolism, which would result in reduced fat deposition and the observed relatively smaller body sizes (Fig. 1A). These results contrast with those of similar experiments in which inoculation of mosquitoes with Escherichia coli reduced the expression of fatty acid catabolic genes in mosquito larval midguts (10). Likewise, separate introductions of Bacteroides thetaiotaiomicron and Butyrivibrio fibrisolvens were correlated with increased fat deposition in the body and restored energy homeostasis in mice (30, 36, 43). The majority of the commensals used in polyspecific inoculation were members of the *Bacteroidetes* (*n* = 11), four of which were *Bacteroides* spp. that shared many orthologs and metabolic pathways observed in *B. thetaiotamicron*, including short-chain fatty acid biosynthesis and dietary carbohydrate degradation (33). Nonetheless, these commensals were not capable of supporting normal development and elicited what is being interpreted as an energy stress response in *P. americana*. Finally, it was notable that introduction of these commensal bacteria into GF insects not only did not decrease the overexpression of endogenous glycosyl hydrolases in the midgut of *P. americana* but elicited the overexpression of additional isoforms (Fig. 5). The *P. americana* bacterial commensals used in GN insects are highly polysaccharolytic organisms (33) and may be in competition with the host for dietary starches. All isolates could colonize hindgut and, except for *Bacteroides* sp. strains PAB214 and PAD25, midgut tissues of both Conv and GN insects (Fig. 6A and B). The three most abundant isolates (i.e., PAD511, PAB519, and PAD521) (Fig. 6A and B) that colonized the GN midgut are highly amylolytic bacteria (33). This suggests that prominent members of the gut community may be competing with the host for this resource (i.e., starch). In the hindgut, introduction of these commensals reduced expression levels of several endogenous glycosyl hydrolases to, or even below, that observed in conventional insects (Fig. 5). In cockroaches, nonrecalcitrant polysaccharides (e.g., starch) are digested in the midgut while recalcitrant fibers like cellulose and pectin are hydrolyzed in the hindgut by their microbiota (44). Interestingly, cellulolytic members of *Bacteroidetes* were in higher abundance in the GN hindgut (Fig. 6B). Given that *Bacteroidetes* and *Firmicutes* are frequently more abundant and typically domiciled in the hindgut and can hydrolyze many complex carbohydrates, it is possible that these commensals could contribute to diet processing and surpass a more normative host response.

Although successful gut tissue colonization by, and the genomic capabilities of, the selected isolates predict their potential positive contribution to dietary nutrient availability, they alone were incapable of restoring normal growth and developmental phenotypes in *P. americana*. These data suggest that *P. americana* relies upon specific commensals, which is distinct from results from some other invertebrate models. Coprophagy is commonly practiced by cockroaches and termites and is thought to provide a reliable means for acquiring, and reacquiring following molting events, gut commensals throughout their lives (45). *P. americana* gut commensal species diversity is at least 2 orders of magnitude greater than that of honeybees, mosquitoes, and fruit flies, and thus the subset of isolates used to inoculate the gnotobiotic insects may have been insufficient to provide the as-yet-characterized metabolites and/or services required by the host for normal development. Further investigation is necessary to ascertain which commensals can restore typical host phenotypes, as microbiome status was the single variable between the treatments in this study.

### Conclusions.

Elimination of normal gut microbial communities in several invertebrate and vertebrate animal models yields bacterium-associated physiological and developmental phenotypes that can illustrate how microbes and their hosts interact and have shaped one another. Germfree *P. americana* insects exhibit transcriptional, morphological, and developmental bacterium-associated phenotypes that reflect energy/nutrient stress, as observed in other animals that include mice, mosquitoes, honeybees, and fruit flies. Rearing *P. americana* insects independently of their normal gut commensals resulted in dramatic alterations in their normal developmental rate and overall growth, which recapitulates previous work in *P. americana* (19) and reflects outcomes observed in *Drosophila*, *Apis*, and mice (2, 3), maintained under germfree conditions, and highlights how gut microbiota influence several aspects of their host’s life history (46). Gene networks that sense and respond to available nutrients and help coordinate growth and development had altered expression patterns in germfree *P. americana* insects. Orthologous genes in other animals reared under germfree conditions exhibited similar expression patterns, which suggest metazoan-level bacterial integration. Introduction or reassociation of germfree hosts with bacterial species that resolve developmental and physiological bacterium-associated phenotypes is critical toward establishing how hosts and their microbes are connected. In this work, *P. americana* was unique among legacy germfree model systems in that reassociation of germfree insects with a subset of gut commensals did not recover phenotypes observed in conventionalized insects, suggesting that a specific microbial assemblage was required for normal growth and development. It was notable that reassociation of germfree *P. americana* with these bacterial commensals appeared to ameliorate some of the effects of germfree rearing (i.e., reduced expression of *de novo* polysaccharolytic genes) in the hindgut tissues while magnifying other effects in the midgut tissues. As these bacteria are typically abundant and domiciled in *P. americana* hindguts, these results may reflect a tissue-specific, local adaptation, but further work is needed to show if this commensal community could complement functions provided by the more-speciose conventional gut community.

## MATERIALS AND METHODS

### Insects.

*P. americana* nymphs, adults, and ootheca were obtained from a general use population maintained at 30°C in The Ohio State University Biological Sciences Greenhouse insectary in Columbus, Ohio. All insect treatment populations were reared and maintained in a growth chamber under a 24-h dark cycle at 30°C and 60% relative humidity and provided with gamma-irradiated rat chow and access to autoclave-sterilized Milli-Q water (MQW) *ad libitum* (20).

### Germfree *P. americana*.

Germfree insects were generated by surface sterilization of fully or near fully sclerotized ootheca (egg cases) obtained from gravid *P. americana* females, and juveniles were isolated, hatched, and maintained under sterile conditions in sterile rearing chambers according to methods described in detail in reference 19. Each rearing chamber was stocked with sterile rat chow and aseptic 1% agar, sufficient for 60+ days of feeding, and chambers were ventilated with sterile-filtered air via a 0.22-μm syringe filter. This procedure yielded up to 16 germfree (GF) nymphs per oothecum. To account for possible genetic variation between ootheca, up to four first-instar nymphs from each oothecum were included in each of the three treatment populations, and treatment-defined siblings were reared together. Quality control measures employed to affirm the germfree status of insects included nutrient medium-based cultivation and diagnostic PCR of post-surface sterilization oothecum rinses and homogenates of ootheca following nymphal emergence and inspection of DAPI (4′,6-diamidino-2-phenylindole)-stained frass and gut contents from GF nymphs; all quality control approaches are described in detail in reference 19. Any insects associated with positive cultivations, amplification, and/or identification of putative DAPI-stained bacteria were euthanized and excluded from the experiment.

### Gnotobiotic *P. americana*.

First-instar GF insects were inoculated with a defined microbial community comprised of 11 *Bacteroidetes* (33) and two *Firmicutes* isolates recovered from *P. americana* gut homogenates (see Table S3 in the supplemental material) to generate gnotobiotic (GN) *P. americana* insects. Overnight cultures of individual bacterial isolates were grown under anaerobic conditions in modified tryptone yeast glucose (MTYG) liquid medium at 30°C (33). Cultures were normalized to ∼0.9 optical density at 600 nm (OD_600_), and aliquots were collected under anaerobic conditions (90% N_2_, 5% CO_2_, and 5% H_2_) and centrifuged on a benchtop centrifuge for 5 min to pellet cells. Two-thirds of the supernatant was removed, and the cells were resuspended. Cultures were combined in equal ratios, and 20 μl was drawn into 50-μl capillary tubes and topped with sterile mineral oil to prevent air intrusion from the upper end. Water-deprived GF insects were exposed to a mixed defined community by inserting capillary tubes through the sterile habitat wall. Insects were denied access to water to encourage consumption of culture, which occurred within 2 min. Culture was renewed daily for 4 days. After capillary removal, habitat penetrations were sealed with cyanoacrylate and nymphs were provided access to 1% sterile agar for hydration and gamma-irradiated rat chow *ad libitum*.

10.1128/mSystems.00802-21.3 TABLE S3 *P. americana* gut bacterial isolates used to produce gnotobiotic (GN) insects and primers used for their detection in GN and conventionalized (Conv) insects. Download Table S3, DOCX file, 0.01 MB. Copyright © 2021 Vera-Ponce de León et al. 2021 Vera-Ponce de León et al. https://creativecommons.org/licenses/by/4.0/ This content is distributed under the terms of the Creative Commons Attribution 4.0 International license.

### Conventional *P. americana*.

First-instar GF insects were deposited into an aquarium containing 10 adult male *P. americana* insects from a nonsterile mixed-generation colony, and nymphs from this colony were designated “conventionalized” (Conv). Conv hatchlings were free to interact with adult cockroaches and their frass, which encouraged normal coprophagic behavior that led to the acquisition of typical gut commensals. As cannibalism of deceased nestmates is also a putative mechanism for gut microbiota acquisition, late-stage nymphs from the nonsterile colony were sacrificed and deposited in the Conv colony.

### Instar duration and morphometrics.

Nymphs were collected as they molted to fifth instar, and the duration (days) to this instar was recorded. Previous work has shown that the cumulative effects of bacterial colonization, or lack thereof, in the host could be observed in fourth and fifth instars (19), and thus all experimental measures in this study were taken from fifth-instar individuals. Nymphs were dissected approximately 5 days after molting, at peak of feeding within instar (47), to minimize variability in gut morphology and gene expression associated with instar transitions. Five morphological metrics were collected, measured in millimeters unless otherwise noted: body length, body width, body mass (in grams), midgut length, and hindgut length. The FIJI image analysis package (48) was used to perform measurements of the dissected guts and their corresponding carcasses from digital images of these tissues taken immediately prior to and following dissection. Additionally, gut compartments and bodies were traced, and lengths were measured. All measurements were specifically calibrated to a scale in each photo obtained. Mass measurements were also collected for whole GF, GN, and Conv individuals prior to dissection by use of a microbalance. Differences between GF, GN, and Conv body measurement parameters were evaluated by a Kruskal-Wallis test, followed by a Dunn test for pairwise comparisons, and Bonferroni correction-adjusted *P* values were reported. All statistical analysis was conducted using R.

### RNA extraction and purification.

Individuals in each treatment population were collected and weighed at fifth nymph instar, cold-anesthetized insects were superficially rinsed with phosphate-buffered saline (PBS), and the total gut including Malpighian tubes was dissected under a stereomicroscope using sterile fine forceps. Gut tissues were embedded in RNAlater stabilization solution (ThermoFisher), and Malpighian tubes were removed. Gut tissues were sectioned from 30 individuals into midgut and hindgut sections and pooled in three batches of 10 and stored in 200 μl RNAlater at 4°C. Total RNA was collected from these pools using the RiboZol RNA extraction reagent (VWR) and cleaned with the PureLink RNA minikit (ThermoScientific) after on-column DNase digestion in accordance with the manufacturer’s procedure. Pure RNA was collected in 70 μl RNase-free water, quantified by a Nanodrop, and visualized on a 1% electrophoresis gel. RNA integrity was analyzed on an Agilent 2200 TapeStation according to the manufacturer’s recommendations. Poly(A) mRNA was filtered using the NEBNext poly(A) mRNA magnetic isolation module according to the manufacturer’s instructions (NEB). cDNA libraries were generated with the Ultra II directional RNA library kit (NEB). All samples were sequenced using an Illumina HiSeq 4000 sequencer (Illumina) with an average of 20 million paired-end reads (2 × 150 bp) at The James Cancer Center sequencing facilities (The Ohio State University, Columbus, OH, USA).

### Transcriptome assembly, annotation, and analyses.

FastQC v.0.11.7 (https://www.bioinformatics.babraham.ac.uk/projects/fastqc/) was used for a quality check of all raw reads, and those with a “phred” quality score below 30 and Illumina adaptors were removed from sequences by TrimGalore v.0.4.5 (https://www.bioinformatics.babraham.ac.uk/projects/trim_galore/; –illumina -q 30 –retain_unpaired –paired parameters). Only reads that mapped to the publicly available *P. americana* genome (49) were used in these analyses to discard possible contaminants. High-quality reads from each treatment were mapped against the *P. americana* genome (49) (GenBank accession no. PGRX00000000.1) using Hisat2 v.2.1.0 (50) (parameters -p 8 -x P.americana.genome.index -1 <Tissue>.<replicate > .1.fq -2 <Tissue>.<replicate > .2.fq -S <file>.sam), and all “sam” files were converted to “bam” files with SAMtools 1.6 (51). Paired mapped reads were retrieved by the fastq tool from SAMtools (parameters SAMtools fastq -@ 8 -f 2 -1 <Tissue>.<replicate > .1.mapped.fq -2 <Tissue>.<replicate > .2.mapped.fq <file>.bam), and these mapped paired reads were used for *de novo* transcriptome assemblies using Trinity v.2.5.0 (52) (parameters: Trinity –SS_lib_type RF –seqType fq –max_memory 900G–SS_lib_type RF –seqType fq –max_memory 900G–SS_lib_type RF –seqType fq –max_memory 900G –CPU 48 –full_cleanup). Transcriptome assembly completeness was estimated using the BUSCO v.3.2.0 (53) pipeline (parameters: -m trans -l insecta_odb9 -c 16 parameters) using the insecta_odb9 orthologue database. All scripts for mapping and assembly were deposited in GitHub. Protein coding sequences were retrieved from assembly transcripts with Transdecoder v.5.2.0 (https://github.com/TransDecoder), and putative functional domains and proteins in translated transcripts were annotated by BLAST (54) and HMMR (55) searches of UniRef90 and PFAM. All BLAST and HMMER tables were merged and sorted using the Trinotate pipeline (56), and all transcripts were also used to BLAST search a custom database comprised of all invertebrate genomes in NCBI (September 2018). All transcripts with at least one annotation resulting from either Trinotate or invertebrate genome database searches were included in the final transcriptome annotation. All transcripts matching sequences assigned to bacteria were excluded from further analyses.

Transcriptomes were defined by treatment (germfree [GF], gnotobiotic [GN], or conventionalized [Conv]) and gut tissue (midgut [M] or hindgut [H)] for differential expression analyses. Counts of high-quality transcriptome sequencing (RNA-Seq) reads from each treatment mapping to Trinity-assembled genes and isoforms (Table 1) were quantified using Salmon v.0.9.1 (57) with the following parameters: quant -p 16 -i transcripts_index -l ISR -1 <Tissue>.<replicate>.R1_val_1.fq -2 <Tissue>.<replicate>.R2_val_2.fq -o <Tissue>.<replicate>_transcripts_quant. A total transcript count matrix was generated using the tximport Bioconductor (58) library in R. DESeq2 (59) was used to detect differentially expressed isoforms and genes by using, as a comparison, each treatment in midgut and hindgut tissues. Isoforms/genes were considered differentially expressed if they showed a false discovery rate (FDR) (Benjamini-Hochberg multiple test correction [60]) value of ≤0.05 and an absolute fold change of >1.5. Principal-component analysis (PCA) plots were used to evaluate gene expression patterns generalized across tissue and/or bacterial presence and performed by the DESeq2 “plotPCA” function with the variance stabilizing transformation (vst) method. To further quantify the effect on the expression patterns of the *P. americana* transcriptome across tissues (midgut and hindgut) and microbial status (i.e., GF, GN, and Conv), an analysis of variance (ANOVA) test of the expression values of all expressed isoforms was performed using the following R “lm” function: lm(DESeq2_Norm_expression_values ∼ GutRegion + MicrobiomeStatus). The significance and *P* values of this linear model were then calculated using the “anova” function in R.

A hierarchical clustering (HC) analysis was performed to identify genes and isoforms whose expression was influenced by both microbiome elimination and gnotobiosis, and all differentially expressed isoforms from the DESeq2 comparisons analyzed (i.e., GF versus GN, GF versus Conv, and GN versus Conv), in both midgut and hindgut, were examined. The Z-scores of the log_2_ DESeq2 normalized expression values of these isoforms were manually obtained. Z-score values of each isoform were then clustered using the “clusterboot” function from the Flexible Procedures for Clustering (fpc) package in R (https://cran.r-project.org/web/packages/fpc/index.html) with the following parameters: clusterboot(data_z_score, clustermethod=kmeansCBI, k = 5, seed = 100). This function was used to perform both hierarchical clustering and bootstrap analysis of all clusters generated. Stability in the clusters was then evaluated by analyzing the Jaccard similarity coefficient values in each cluster as was established by Hennig (20). All clusters were summarized and visualized in a heat map by the “pheatmap” function in R (https://cran.r-project.org/web/packages/pheatmap/index.html). Functional enrichment and gene ontology (GO) categories in each cluster from the hierarchical clustering analysis were obtained using GOseq (61). All assembled transcripts showing normalized expression values above 0 were selected as a background. GO ancestries for each isoform were extracted from the Trinotate annotation table using the extract_GO_assignments_from_Trinotate_xls.pl script (https://github.com/Trinotate/Trinotate.github.io/wiki). GO enrichment categories in each cluster were obtained using the run_GOseq.pl from Trinity v.2.6.6 (52) (parameters: run_GOseq.pl –factor_labeling <Cluster.factor> –GO_assignments GO.transcripts.ancestry.tab –lengths isoform.length.txt –background Background.isoforms). GO categories with a false discovery rate (FDR) below 0.05 after the Benjamini and Hochberg correction procedure (60) were identified as significantly enriched, and these were clustered, summarized, and visualized using the REVIGO (62) tool, with an allowed similarity of 0.50 as a summarizing cutoff.

### Classification of differentially expressed *P. americana* genes/isoforms involved in nutrition, development, and tissue homeostasis in gut tissues under GF, GN, and Conv treatments.

Protein coding sequences of genes associated with the insulin/insulin-like (IIS) growth factor, target of rapamycin (TOR), Hippo, and AMPK pathways were retrieved from the public *P. americana* genome annotation (49). For this, all gut transcriptome isoforms were aligned to the *P. americana* protein coding sequences using BLASTx searches. All BLAST hits with identity values higher than 70% and E values less than 0.0005 were considered for further analysis. Additionally, the expression values of the isoforms associated with programmed cell death (GO:0012501), carbohydrate utilization (GO:0009758), and fatty acid catabolic process (GO:0009062) were retrieved from the Trinotate annotation tables using custom R scripts. The isoforms within these pathways with a fold change of  ≥1.5 and an FDR of ≤0.05 were considered differentially expressed. Heat maps for each specific metabolic pathway were used to visualize all differentially expressed isoforms between GF, GN, and Conv comparisons.

### Quantification of colonizing bacteria used for gnotobiosis in GN and Conv *P. americana* midgut and hindgut.

Bacterial colonization of fifth-instar GN gut tissues was quantified by RT-qPCR using the same RNA samples extracted from midgut and hindgut tissues that were used for the transcriptome profiling experiments (see above). Standard curves were generated using a 192-bp plasmid-bound 16S rRNA gene fragment reference diluted to 10^3^ to 10^9^ plasmid copies per microliter. Isolates were amplified with corresponding isolate-specific primers designed (Table S4) to generate ∼200-bp amplicons, and two technical replicates were conducted for each biological replicate in a 10-μl PCR mixture. Isolate-specific primers were searched against the bacterial Arb-SILVA 16S rRNA database (release 128) and against an in-house bacterial 16S rRNA database of cockroach gut isolates to ensure specificity. Isolate-specific primers were further tested against closely related species within an in-house isolate collection to ensure amplification specificity. RT-qPCR utilized Thermo Scientific Verso 1-step 2× master mix, and thermocycler conditions were as follows: 50°C for 15 min, 95°C for 15 min, followed by 35 cycles of 1 min at 95°C, 15 s at 58°C, 40 s at 68°C, and 10 s signal acquisition at 78°C. A final melt curve analysis was performed to differentiate target amplification from primer dimer. 16S rRNA count data were normalized by sample RNA concentration.

10.1128/mSystems.00802-21.4 TABLE S4 Primer used for bacterial detection in GN and Conv insects. Download Table S4, DOCX file, 0.01 MB. Copyright © 2021 Vera-Ponce de León et al. 2021 Vera-Ponce de León et al. https://creativecommons.org/licenses/by/4.0/ This content is distributed under the terms of the Creative Commons Attribution 4.0 International license.

### Data availability.

RNA-Seq raw data, transcriptome assemblies, and annotations have been deposited in the National Center for Biotechnology Information Gene Expression Omnibus (NCBI-GEO) database under accession number GSE159954, with links to BioProject accession number PRJNA666210. A description of all software, including scripts and commands, used for the analyses in this article can be found at https://github.com/avera1988/P.americana_RNAseq.
