# Supplementary material for: Microbiota Perturbation or Elimination Can Inhibit Normal Development and Elicit a Starvation-Like Response in an Omnivorous Model Invertebrate
Source: mSystems. 2021 Aug 24;6(4):e00802-21. doi: 10.1128/mSystems.00802-21 (PMC8407121; doi:10.1128/mSystems.00802-21)
Supplement: TABLE S1 [file msystems.00802-21-st001.docx]

**Table S1. General features of *P. americana* genome and gut transcriptome.**

| **Feature** | **Value** |
| --- | --- |
| Total genome size***** | 3.38 Gb |
| Protein coding genes***** | 21, 336 |
| Gut transcriptomic assembled | 325 Mb |
| Gut transcriptomic completeness****** | 93.6 % |
| Total Trinity transcripts | 553,147 |
| Total of annotated transcripts | 65,047 |

* Data from (1)

** Based on BUSCO analysis using the *Insecta* orthologs database.

1. S. Li, *et al.*, The genomic and functional landscapes of developmental plasticity in the American cockroach. *Nat. Commun.* **9** (2018).
