# Supplementary material for: Microbiota Perturbation or Elimination Can Inhibit Normal Development and Elicit a Starvation-Like Response in an Omnivorous Model Invertebrate
Source: mSystems. 2021 Aug 24;6(4):e00802-21. doi: 10.1128/mSystems.00802-21 (PMC8407121; doi:10.1128/mSystems.00802-21)
Supplement: TABLE S2 [file msystems.00802-21-st002.docx]

**Table S2. Summary of the analysis of variance (ANOVA) on the expression values of differential expressed transcripts in the *P. americana* midgut and hindgut (gut region) under three different microbiome status conditions (i.e. germ-free, gnotobiotic and conventionalized).**

|  | DF | Sum sq | Mean sq | F value | P value |
| --- | --- | --- | --- | --- | --- |
| Gut region | 1 | 4.52e^6^ | 4.52e^6^ | 1.46 | 0.23 |
| Microbiome status | 2 | 8.60e^7^ | 4.30e^7^ | 22.82 | 9.11e^-7^ |
