# Supplementary material for: Microbiota Perturbation or Elimination Can Inhibit Normal Development and Elicit a Starvation-Like Response in an Omnivorous Model Invertebrate
Source: mSystems. 2021 Aug 24;6(4):e00802-21. doi: 10.1128/mSystems.00802-21 (PMC8407121; doi:10.1128/mSystems.00802-21)
Supplement: TABLE S3 [file msystems.00802-21-st003.docx]

**Table S3. *P. americana* gut bacterial isolates used to produce gnotobiotic (GN) insects and primers used for their detection in GN and conventionalized (Conv) insects.**

| Taxa | Isolate Name | Primer name | Direction | Sequence |
| --- | --- | --- | --- | --- |
| *Dysgonomonas* | PAD521 | PH5-21.655 R | reverse | CTCAAGGCTACCAGTTTCAACGG |
|  | PAD521 | PH5-21.464 F | forward | CATTACGTGTAGTGTATTGCATGTACTG |
|  | PAD520 | PH5-20.653 R | reverse | AAGTCTTCCAGTTTCAACGGCAA |
|  | PAD520 | PH5-20.445 F | forward | GTGCTAGGGTAAAACATATCACGAGTG |
|  | PAD511 | PH5-11.673 R | reverse | CCGCCTACTTCATCTATACTCAAGAAAC |
|  | PAD511 | PH5-11.463 F | forward | TACGTGTAGTATATTGCATGTACCATATG |
|  | PAD25 | 25.23S.2_F | forward | CTCCGAATGGGTACAAGGGTCAT |
|  | PAD25 | 25.23S.2_R | reverse | TCCATGCAGAACCACTCGACTAG |
|  | PAD216 | PFB2-16.648 R | reverse | AACCCAGTTTCAACGGCAATTTTAAG |
|  | PAD216 | PFB2-16.444 F | forward | GTACTAGGGTAAAACAGGGGACGT |
| *Paludibacter* | PAR221 | PFB2-21.667 R | reverse | GCCTCTACTGCACTCAAGAACAC |
|  | PAR221 | PFB2-21.476 F | forward | TTGTATGTACTTTACGAATAAGCATCGG |
| *Parabacteroides* | PAP52 | PF5-2.654 R | reverse | TCAAGACTAACAGTTTCAACGGCA |
|  | PAP52 | PF5-2.435 F | forward | CTTCTTTTATTGGGGAATAACGGCAG |
| Lachnospiraceae | PAL227 | PFB2-27.64 F | forward | AAGCACTTCTCCCGATGAAGCAAG |
|  | PAL227 | PFB2-27.262 R | reverse | ACGCGGGTCCATCTCATACC |
|  | PAL113 | PFB1-13.56 F | forward | CGAAGCATTTCTTTTGGAAGCGAT |
|  | PAL113 | PFB1-13.262 R | reverse | ACGCGGGTCCATCTCATACC |
| *Bacteroides* | PAB519 | PH5-19.682 R | reverse | CGAATTCCGCCAACCTTTACTTTAC |
|  | PAB519 | PH5-19.451 F | forward | TGGGAATAAAGTGACGTACGTGT |
|  | PAB51 | PH5-1.670 R | reverse | ACCTCCACTATACTCAAGACGCC |
|  | PAB51 | PH5-1.467 F | forward | CACGTGTGGAGTTTTGTATGT |
|  | PAB224 | PAB224rDNAintergen_F | forward | TCAAGAAGCCGAGCCGTAAG |
|  | PAB224 | PAB224rDNAintergen_R | reverse | GCTGAGCTAATCCCCCGATAAG |
|  | PAB214 | PFB2-14.677 R | reverse | TCCGCCTACCTCTACTGTACTCA |
